# Short Neuropeptide F and Its Receptor Regulate Feeding Behavior in Pea Aphid (*Acyrthosiphon pisum*)

**DOI:** 10.3390/insects13030282

**Published:** 2022-03-13

**Authors:** Muhammad Bilal Amir, Yan Shi, Hehe Cao, Muhammad Yasir Ali, Muhammad Afaq Ahmed, Guy Smagghe, Tong-Xian Liu

**Affiliations:** 1 Key Laboratory of Insect Ecology and Molecular Biology, College of Plant Health and Medicine, Qingdao Agricultural University, Qingdao 266109, China; dr.mbilalamir@outlook.com (M.B.A.); shiyanyuanyi@aliyun.com (Y.S.); caohehe1988@163.com (H.C.); m.yasirali4663@gmail.com (M.Y.A.); afaqahmed011@hotmail.com (M.A.A.); 2 Laboratory of Agrozoology, Department of Plants and Crops, Faculty of Bioscience Engineering, Ghent University, 9000 Ghent, Belgium

**Keywords:** *Acyrthosiphon pisum*, short neuropeptide F, G protein-coupled receptor, transcriptional expression, RNA interference, feeding regulation

## Abstract

**Simple Summary:**

We know that neuropeptides and G protein-coupled receptors regulate the physiology and behavior of animals and that the pea aphid (*Acyrthosiphon pisum*) is a serious agricultural pest and model insect. In this study, we investigated the short neuropeptide F and its receptor in pea aphid. Feeding analysis showed that the probing time and total phloem duration significantly decreased in response to sNPF and predicted sNPFR gene silencing in RNAi assays. The silencing of sNPF significantly reduced the aphid’s reproduction but not survival. Our findings will help in the design of control strategies by using the molecular biological approach.

**Abstract:**

Insect short neuropeptide F (sNPF), an ortholog of prolactin-releasing peptide of invertebrates, regulates diverse biological processes, including feeding, olfaction, locomotion, and sleep homeostasis in insects. However, its function is still unclear in an important model insect and agricultural pest, the pea aphid (*Acyrthosiphon pisum*). Here, we investigated short neuropeptide F (*ApsNPF*) and its receptor (*ApsNPFR*) in *A. pisum*. The sNPF gene contains three exons and two long introns. In addition, the genome contains a single sNPF receptor with seven transmembrane domains. Stage- and tissue-specific transcript profiling by qRT-PCR revealed that *ApsNPF* and *ApsNPFR* were mainly expressed in the central nervous system. The receptor was also detected in antennae, midgut, and integument. The highest expression levels were found in first instar nymphs compared to other developmental stages. Besides, the starvation-induced pattern indicated that the sNPF network depends on the nutritional state of the insect. An electrical penetration graph showed that probing time and phloem duration of *A. pisum* on broad bean plants decreased in response to dssNPF and dssNPFR in RNAi assays. sNPF silencing reduced the number of nymphs per female but not aphid survival. We believe that our results advance in-depth knowledge of the sNPF/sNPFR signaling cascade and its place in regulating feeding behavior in insects. In turn, it may contribute to the potential design of new strategies to control aphids, with a focus on the sNPF system.

## 1. Introduction

Neuropeptides regulate a wide array of behavior and physiological processes in arthropods, including feeding, molting, courtship, social interaction, and development. They play their role by binding as ligands with cognate G protein-coupled receptors (GPCRs), which initiates the signaling process [1,2]. Feeding is a complex behavior present in all species, and it is modulated by environmental clues and internal processes. For survival, animals coordinate internal and external cues, and they act in ways that maintain energy homeostasis centrally and peripherally and ensure proper nutrition intake [3].

The discovery of short neuropeptide F occurred when a specific antibody against the neuropeptide F of *Moniezia expansa* was applied to assay the similar peptide in insects. Interestingly, the result was the isolation of a novel neuropeptide in *Leptinotarsa decemlineata* [4]. These peptides were identified by the use of NPF antibodies, called NPF-related peptides. They were composed of a short chain of 8-12 amino acids compared to NPF (36-42 amino acids), so they were renamed short neuropeptide F (sNPF) [5]. The cognate receptor for short neuropeptide F (sNPF) was first documented in *Drosophila melanogaster*. It belongs to the superfamily of GPCRs and exhibits 62–66% similarity and 32–34% identity with vertebrate type 2 NPY receptors [6].

Peptides that pertain to the sNPF lineage are highly conserved and characterized mainly by xPxLRLRFamide, whereas some insects (flies and mosquitoes) present a modified RWamide sequence at the carboxy-terminal [7]. Until now, sNPF peptide and its receptor have been demonstrated in all model and representative insects, such as *D. melanogaster* [8,9], *Bombyx mori* [10,11], *Schistocerca gregaria* [12,13], *Anopheles gambiae* [14], and *Tribolium castaneum* [15]. sNPF peptide acts as a neuromodulator and is attributed to a variety of physiological functions, such as memory and olfaction [16,17], locomotion [18], reproduction and survival [19,20], hormone release or suppression [9,21], energy homeostasis [22], and sleep and circadian rhythms [23,24,25,26]. However, the primary function of sNPF signaling is in feeding either regulatory [10,27,28,29,30] or inhibitory [12,13,14,31].

Although sNPF peptides have been widely studied in numerous insects, their localization and function in *A. pisum* is still unknown. The pea aphid is an important phloem sapsucking model insect, and it is also a notorious agricultural pest that mainly targets horticultural crops and causes significant yield losses [32]. Moreover, in various studies, *A. pisum* is regarded as a model insect due to its host adaptability, parthenogenesis, and polyphenism [33].

To study the function of *ApsNPF* and *ApsNPFR* and whether there is a relationship between sNPF signaling and feeding, we investigated the transcripts of both genes in different developmental stages, tissues, and under the condition of induced starvation stress. Furthermore, we knocked down these genes in RNAi assays and studied the feeding behavior through an electrical penetration graph (EPG) approach. We also analyzed the influence of sNPF silencing on physiological attributes, such as reproduction and survival. We believe that our results may advance our current knowledge of the sNPF/sNPFR signaling cascade and its place in the regulation of feeding behavior in insects. In turn, this may contribute to the potential design of new strategies to control aphids, with a focus on the sNPF system.

## 2. Materials and Methods

### 2.1. Insect Rearing 

We experimented with the green strain of parthenogenetic *A. pisum* derived from a long-established apterous population at our laboratory (initially brought from Ghent University, Belgium). Insects were reared on 3–4-week-old broad bean (*Vicia faba*) seedlings in an environment-controlled incubator (Jiangnan, Ningbo, China) at a standard condition of 20 ± 1 °C, 70 ± 5% RH and an 16:8 h (light:dark) photocycle. Aphids were maintained at a low density of ~5 insects per plant in order to prevent the development of a winged population and competition. The nymphs aged 0–12 h were collected and deposited on fresh broad bean leaves to obtain synchronously developed insects.

### 2.2. Identification of Sequence and Phylogenetic Analysis

From the published literature on *A. pisum*, neuropeptide and neurohormone precursors [34], as well as GPCRs [35], cDNA sequences encoding *ApsNPF* and *ApsNPFR*, were obtained. The open reading frame (ORF) for precursor and receptor were confirmed through the ORF finder (https://www.ncbi.nlm.nih.gov/orffinder/, accessed on 7 February 2020). For precursor, the signal peptides were predicted using the SignalP-5.0 server (http://www.cbs.dtu.dk/services/SignalP/, accessed on 8 February 2020), and the sequence logo of the C-terminal motif of *ApsNPF* was made using Weblogo [36]. For the receptor, transmembrane segments were predicted using the TMHMM server (http://www.cbs.dtu.dk/services/TMHMM/, accessed on 9 February 2020). *ApsNPF* and *ApsNPFR* sequence alignments with other precursor and receptor sequences were made with MEGA 5.2 and JalView 2.9, respectively [37,38].

sNPF gene sequences from other arthropod species were obtained using BLAST (https://blast.ncbi.nlm.nih.gov/Blast.cgi, accessed on 8 February 2020). The online tool Splign (https://www.ncbi.nlm.nih.gov/sutils/splign/splign.cgi, accessed on 17 February 2020) [39] was used by submitting the nucleotide accession number of already discovered sNPF genes against whole genome shotgun sequences of target insects to determine and analyze the exon-intron positions. A schematic figure illustrating the sNPF gene structures was created using IBS 1.0 [40]. A phylogenetic tree was made by the selection of conserved domain regions for *ApsNPF* and *ApsNPFR*, among other species, via ClustalX2 software with the default settings: a neighbor-joining approach, followed by 1000 bootstrap tests, *p*-distance model with a pairwise deletion in MEGA 5.2 [37].

### 2.3. Stage- and Tissue-Specific Expression Profile of A. pisum sNPF/sNPFR via qRT-PCR

The transcript expression levels of *ApsNPF* and *ApsNPFR* were quantified in different developmental stages by collecting 20 aphids from each instar separately in 1.5 mL Eppendorf Safe-Lock tubes (Life Science, Hamburg, Germany), quickly frozen in liquid nitrogen (LN2), and stored at −80 °C. Likewise, samples were prepared to investigate the transcript distribution of these two genes in various tissues. Hereto, 200 wingless adult aphids were dissected carefully in chilled 0.01 M PBS under a stereomicroscope (Olympus, Tokyo, Japan). Subsequently, antennae, central nervous system (CNS), embryos (embryos chain), midgut, and integument were collected and immediately stored as mentioned above. A Bullet Blender Blue (Next Advance, New York, NY, USA) was used to homogenize tissues before RNA extraction. Total RNA was extracted using TRIzol reagent (Invitrogen, Carlsbad, CA, USA) and purified through with an RNeasy mini kit (Beijing, China). The concentration and quality of resultant RNA were measured on a NanoDrop 2000 spectrophotometer (Thermo Fisher Scientific, Waltham, MA, USA). cDNA was prepared using a PrimeScript RT reagent kit with gDNA Eraser (Takara, Kusatsu, Japan). The resulting cDNA template was used to perform a quantitative (real-time) reverse transcription-polymerase chain reaction (qRT-PCR).

Appropriate gene-specific primers were designed using an online program, Primer 3 (http://bioinfo.ut.ee/primer3-0.4.0/, accessed on 3 March 2020), to assay the relative expression level via qRT-PCR (Table S1) and obtained from Sangon Biotech (Shanghai, China). To ensure the accuracy and stability of all samples, a melting curve analysis from 55 to 95 °C was conducted for all reactions. The specificity of each primer set was confirmed by the melting curve, which showed only one peak that was gene-specific, and the linear standard curve was used to determine the efficiency of amplification (E value) using the equation E = 10 − 1/slope. The resultant efficacy was >90%. qRT-PCR was performed on a LightCycler^®^ 96 instrument (Roche, Basel, Switzerland). The reaction mix consisted of 5 μL of TB Green^®^ Premix Ex Taq II (Takara, Japan), 2 μL of nuclease-free water, 1 μL of each primer (forward and reverse), and 1 μL of cDNA template. The following thermal cycling program was used as standard: an initial denaturation at 95 °C for 30 s, followed by 40 cycles at 95 °C for 5 s and 60 °C for 20 s; at the end, the parameters were modified to 95 °C for 10 s, 65 °C for 60 s, and 97 °C for 1 s. For the reference gene, we chose ribosomal protein RPL7 (NM_001135898.1 [41]) and analyzed the relative quantification of expression by using the 2^−ΔΔCt^ procedure [42]. We conducted three biological replicates and one technical replicate for this experiment.

### 2.4. Transcript Pattern during Feeding and Starvation Stress via qRT-PCR

We investigated whether the fed (received food) and starvation stress states (no food) of aphids correlate with the transcript expression of *ApsNPF* and *ApsNPFR*. Hereto, 60 wingless adult aphids for feeding (control) and an identical group for starvation (treatment) were placed in a clip cage (3.5 × 1.5 cm, d × h) positioned on the ventral side of *V. faba* leaves (three aphids per cage and three cages per seedling). The top opening of the clip cage was closed with a fine cloth net. We used four sheets of fine cloth net inside the cage for the starvation treatment to prevent the aphids from feeding [43]. After 3 and 6 h of starvation stress, we randomly selected four aphids for whole-body RNA extraction and 50 aphids for dissection to obtain CNS. Dissection was carried out carefully under a binocular microscope, and samples were stored at −80 °C.

### 2.5. Double-Stranded RNA Synthesis and Injection

An injection-based RNAi bioassay was performed to explore the function of sNPF signaling in *A. pisum*. The unique nucleotide region of *ApsNPF* and *ApsNPFR* was selected (Table S1) and added with T7 promoter sequence at their 5’ ends, designed using primer 5 (Premier Biosoft, Palo Alto, CA, USA), and green fluorescent protein (GFP) was used as a negative control. The primers were purchased from Sangon Biotech (Shanghai, China) and specified using the polymerase chain reaction. A MiniBEST agarose gel DNA extraction kit (Takara, Japan) was used to extract amplicons from the gel and measure their quantities. Double-stranded RNA was prepared with a TranscriptAid T7 high-yield transcription kit (Thermo Fisher, Vilnius, Lithuania); according to the protocol, its concentration was measured, and then it was immediately kept at −80 °C. Furthermore, the integrity of dsRNA was tested by 1% gel electrophoresis.

After the third day of adult emergence, we randomly chose the insects for injection. Sharp needles (3.5-in 3− 000-203-G/X micropipettes, Drummond Scientific, Broomall, PA, USA) were prepared by a PC-100 dual-stage glass micropipette puller (Narishige, Setagaya-Ku, Tokyo, Japan). We injected dsRNA at 300 nL (~1.02 µg) for sNPF, sNPFR, and GFP. An injection was carried out under a stereomicroscope (Olympus, Tokyo, Japan) between the 2nd and 3rd abdominal segments using a 20 nanoliter injector (World Precision Instruments, Sarasota, FL, USA). Prior to injection, aphids were immobilized on a petri dish containing 1% flexible agarose gel with tiny x-shaped grooves to restrict aphid movement.

### 2.6. Transcript Expression after RNAi

After performing the RNAi bioassay, the insects were transferred in the clip cage without a cloth net between leaf and insect. Insects were collected at 6, 12, 24, 36, 48, and 72 h post-injection (hpi) for whole-body RNA extraction. Four aphids were chosen randomly for each treatment, and three biological repeats were carried out.

### 2.7. EPG Analysis of Aphid Feeding Behavior

The EPG approach was used to compare data on probing and feeding behavior between the dssNPF and dssNPFR treatments and the dsGFP control group [44]. After 12–24 hpi, an electrode of gold wire (2 cm × 18 µm) was glued to the dorsum of randomized aphids by applying electrically conductive silver glue. The wired aphid was positioned on a 3–4-week-old broad bean seedling at the petiole end of the abaxial edge of the topmost developed leaf, and the other side of the electrode was attached to the Giga-8 DC EPG system [45]. The second electrode was inserted into potting soil. The entire experiment was placed in a Faraday cage to protect against electromagnetic interference. EPG recordings started immediately and were monitored for 8 h. The EPG waveform was analyzed using the Stylet+ analysis protocol [43,46]. Subsequent analysis was conducted using the automatic parameter calculation Excel Workbook of EPG data 4.4 [47]. Twenty replicates were performed for each treatment.

### 2.8. Survival and Reproduction Assay

Survival and reproduction assays were conducted to investigate the correlation between sNPF gene silencing and the physiological attributes of aphids. We injected dsRNA into 10 aphids per treatment for positive control and identical for the negative control. The aphids were reared on fresh leaves inside the clip cage, and we recorded the fecundity and mortality of adults continuously after a 12 h duration from the beginning of the RNAi assay. All experiments were performed in the artificial environment box, and three replications were carried out for each treatment.

### 2.9. Statistical Analysis

ANOVA was performed, followed by the least significant difference (LSD) test, to compare the reproduction data and the qRT-PCR data obtained from the spatiotemporal assay of *ApsNPF* and *ApsNPFR*. The data obtained from variation in *ApsNPF* and *ApsNPFR* during feeding and starvation stress and the expression pattern of both genes in response to RNAi-mediated silencing were analyzed through a parametric independent Student’s *t*-test for the comparison of two conditions: treatment and control. We compared the aphid survival data after sNPF silencing via Kaplan–Meier survival log-rank analysis. The feeding behavior data obtained from the EPG recordings were analyzed by ANOVA, followed by LSD (*p* = 0.05), due to its normal distribution. Statistical analysis was conducted employing IBM SPSS 20 (Systat Software, London, UK), and histograms were created using OriginPro 8.5.

## 3. Results

### 3.1. Characterization of ApsNPF and Its Receptor

**Figure 1:**
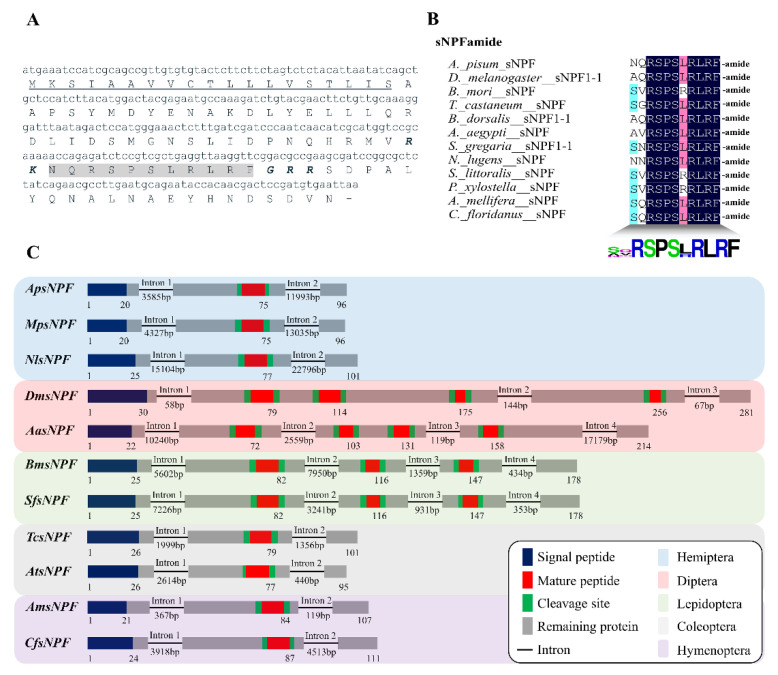
Bioinformatics analysis of short neuropeptide F (sNPF) from *A. pisum*. (**A**) Nucleotide and amino acid sequences of sNPF cDNA. The underlined letters are predicted signal peptides, grey background amino acids represent mature peptides, and italic and bold letters are the dibasic cleavage sites. (**B**) Amino acid sequence alignment of sNPFs with other insect species; at the bottom, there is a consensus logo. (**C**) Comparative analysis of exon/intron structure of the gene encodes *ApsNPF* with sNPF precursors of other insects. The diagram illustrates the gene structure, the rectangles are protein-coding exons, and lines (with the length underneath) are the introns. Species abbreviation: Ap (*Acyrthosiphon pisum*), Mp (*Myzus persicae*), Nl (*Nilaparvata lugens*), Dm (*Drosophila melanogaster*), Aa (*Aedes albopictus*), Bm (*Bombyx mori*), Sf (*Spodoptera frugiperda*), Tc (*Tribolium castaneum*), At (*Aethina tumida*), Am (*Apis mellifera*), and Cf (*Camponotus floridanus*). Table S2 contains the accession numbers of precursors presented in this figure.

First, we confirmed ApsNPF and ApsNPFR cDNA sequences by using gene-specific primers. In gene structural analysis, sNPF contains three exons and two long introns between nucleotides at E28/N29 and Q82/N83 positions (Figure 1C). The sNPF amino acid and nucleotide sequence of the ORF are shown in Figure 1A. An alignment of ApsNPF with sNPF peptides of other insects is presented in Figure 1B, and this reveals that they share the [xPxLRLRFamide] consensus motif at the C-terminal end of the sNPF neuropeptide family.

**Figure 2:**
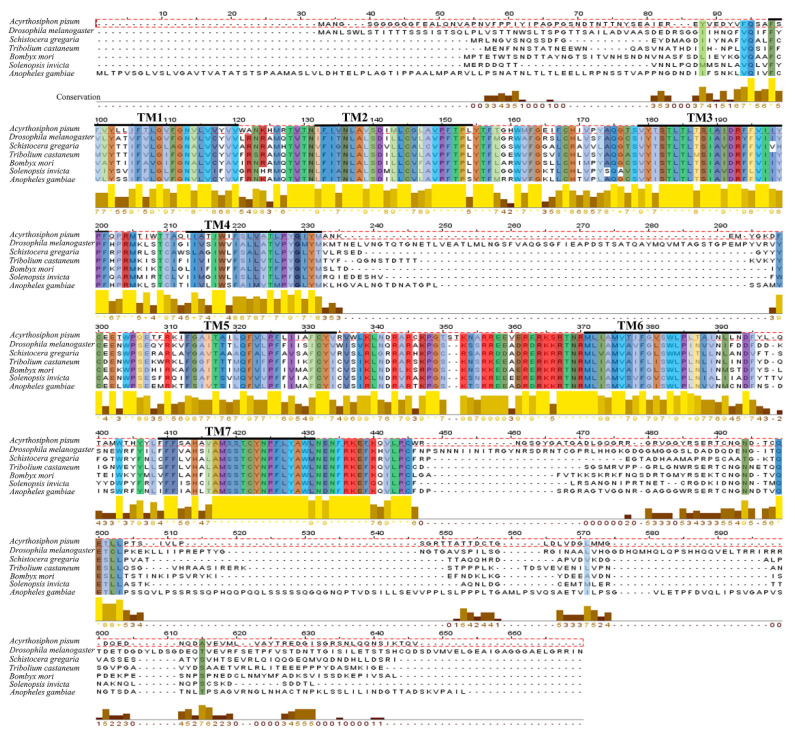
Amino acid multiple sequence alignment of ApsNPFR with sNPFR from other insects (*Drosophila melanogaster*, *Schistocerca gregaria*, *Tribolium castaneum*, *Bombyx mori*, *Solenopsis invicta*, and *Anopheles gambiae*. The degree of similarity is indicated by the height of yellow or brown bars below the sequences. Transmembrane domains are indicated by black horizontal bars (with numbers at the top; TM1–TM7).

**Figure 3:**
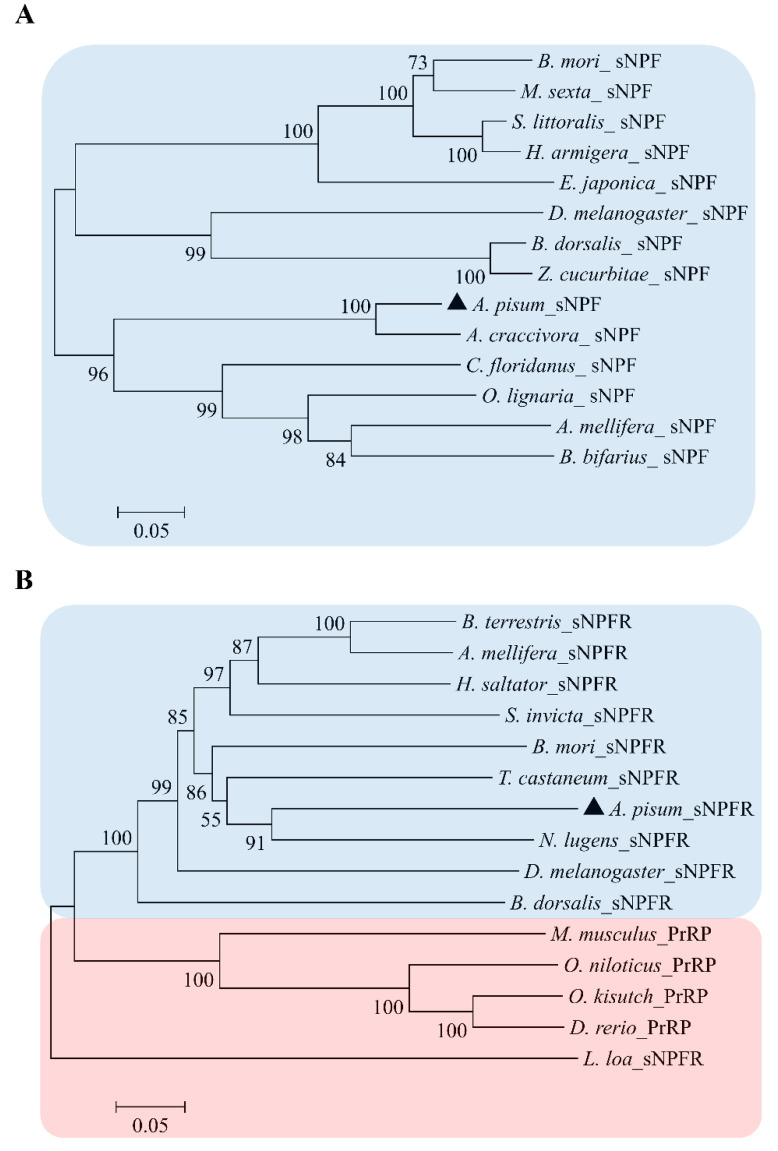
Phylogenetic relationship of sNPF precursors with other insect species (**A**) and sNPF receptors of invertebrates (blue box) with PrRP receptors of vertebrates (red box) (**B**). *Loa loa* sNPFR was selected as outgroup. The percent bootstrap support values are indicated by the number at branches. Table S3 contains the precursor and receptor accession number.

We also confirmed that the genome contains a single predicted receptor for the ApsNPF precursor, which belongs to a typical rhodopsin-like GPCR family with seven alpha-helical transmembrane segments. The sNPFR cDNA sequence includes an ORF of 1374 bp that encodes a protein with 457 amino acids and a predicted MW of 51.44 kD. The ApsNPFR sequence was aligned with other related receptors and revealed a high degree of sequence identity and similarity, with the maximum conservation at the transmembrane regions (Figure 2). Phylogenetic analysis of ApsNPF and ApsNPFR with other insects is presented in Figure 3, and this demonstrates close proximity with other hemipterans, including Aphis craccivora and Nilaparvata lugens.

### 3.2. Stage- and Tissue-Specific qRT-PCR Analysis Shows a Spatiotemporal Transcript Expression of A. pisum sNPF/sNPFR

**Figure 4:**
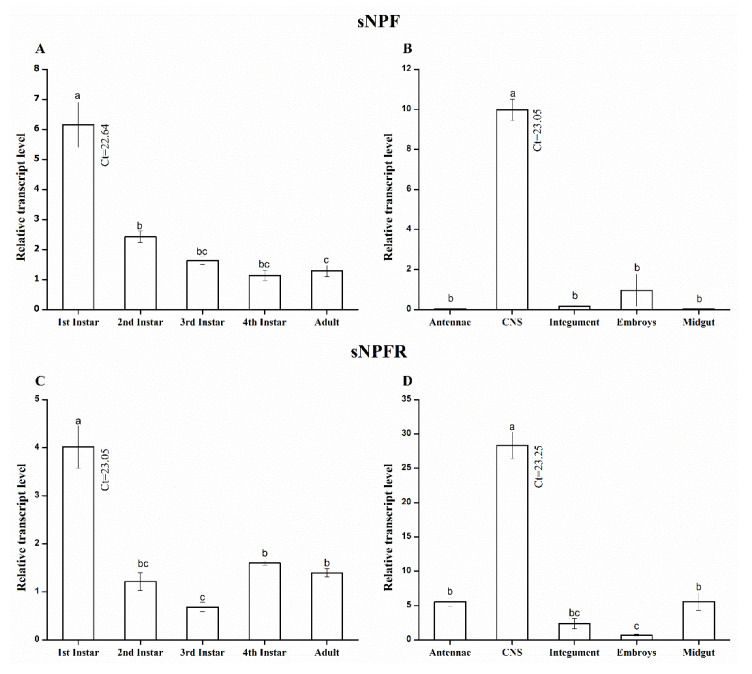
Analysis of relative expression levels of *A. pisum sNPF* and *sNPFR* transcripts measured by qRT-PCR. Development expression profile of *ApsNPF* and *ApsNPFR* (**A**,**C**) and spatial expression profiles of *ApsNPF* and *ApsNPFR* (**B**,**D**). Acronyms used on *X*-axis: CNS (central nervous system), Ct (cycle threshold). The bars correspond to the average of three independent replicates. Results are shown as means ± S.E. Different lowercase letters above each bar indicate significant differences among different treatments using one-way ANOVA followed by LSD (*p* < 0.05).

The relative expression patterns of ApsNPF and ApsNPFR mRNA in A. pisum in different developmental stages and tissues were investigated by qRT-PCR. The transcript profile of these two genes was normalized to the reference gene, RPL7 (Figure 4). The results revealed that ApsNPF is present in all instars, including adults, but the expression level was highest in first instar nymphs (F_4,10_ = 31.01, *p* < 0.001: Figure 4A). Interestingly, a similar pattern was also observed for ApsNPFR. The highest transcript level was detected in the first instar (F_4,10_ = 33.7, *p* < 0.001: Figure 4C), although all life stages exhibited expression of ApsNPFR.

The transcript distribution of ApsNPF and ApsNPFR varied significantly in various tissues. The highest expression of ApsNPF was detected in the CNS (F_4,10_ = 13.23, *p* = 0.001: Figure 4B), and the complete embryos (including the head) also showed some expression. In contrast, ApsNPF was absent (Ct > 30) in antennae, midgut, and integument. For the receptor, the transcript pattern of ApsNPFR was highest in the CNS, but it was also expressed in other tissues, such as antennae, midgut, and integument (F_4,10_ = 101, *p* < 0.001: Figure 4D, Table S5).

### 3.3. Transcript Expression during Feeding and Starvation Stress

**Figure 5:**
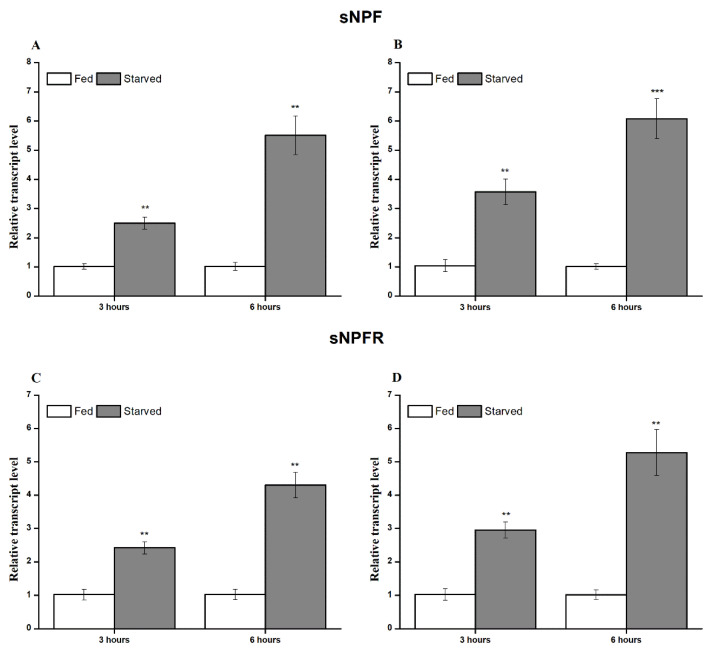
Effect of starvation on the transcript expression of *ApsNPF* and *ApsNPFR* in the adult whole body (**A**,**C**) and central nervous system (CNS) (**B**,**D**). The bars correspond to the average of three independent biological replicates. Results are shown as means ± S.E. Asterisks on bars indicate significant difference between the fed and the starved aphid calculated using statistical analysis (independent student t-test, ** *p* < 0.01; *** *p* < 0.001).

sNPF and its receptor play a crucial role in feeding and nutritional state in numerous insect species. We measured the transcript expression in fed and starved aphids. Both genes showed a significant difference in fed and starved insects. The expression of ApsNPF and ApsNPFR was significantly upregulated in starved aphids in comparison to fed aphids (t_4,3.93_ = 0.11, *p* = 0.001: Figure 5A and t_4,3.12_ = 2.42, *p* = 0.002: Figure 5C, Figure S1).

As the transcripts for ApsNPF and ApsNPFR were primarily detected in the CNS (see Section 3.2), it should be remarked here that we investigated the relative expression in the CNS of starved aphids rather than investigating whole-body expression. Interestingly, we observed the same upregulated expression of sNPF and sNPFR in starved aphids compared to controls (t_4,3.99_ = 0.04, *p* < 0.001: Figure 5B, and t_4,3.99_ = 0.002, *p* = 0.001: Figure 5D, Table S4). Additionally, the transcript expression levels of ApsNPF and ApsNPFR were upregulated with increasing stress of starvation hours.

### 3.4. RNAi-Mediated Silencing of ApsNPF and ApsNPFR via dsRNA Injection

**Figure 6:**
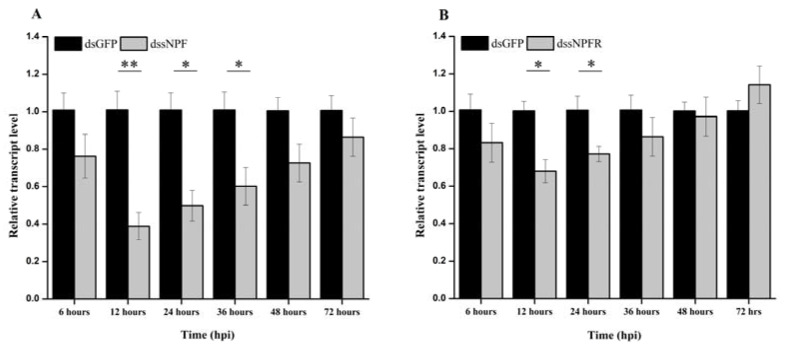
RNAi-mediated knockdown of sNPF (**A**) and sNPFR (**B**) expression levels in *A. pisum*. The transcript patterns in pea aphids injected with sNPF (or sNPFR) dsRNA and GFP dsRNA (control) were measured via qRT-PCR and normalized against RPL7. Acronyms used on X-axis:, hpi (hours post-injection). The bars correspond to the average of three independent biological replicates. Statistical analysis was performed using Student’s t-test (mean ± S.E.; * *p* < 0.05; ** *p* < 0.01).

We investigated the fluctuation in transcript expression of ApsNPF and ApsNPFR via qRT-PCR after RNAi-mediated silencing. The transcript levels of ApsNPF and ApsNPFR were significantly downregulated after 12 h by ~62% and ~32%, respectively (t_4,3.12_ = 2.20, *p* < 0.05: Figure 6A, and t_4,3.2_ = 1.84, *p* < 0.05: Figure 6B, Table S5). The inhibitory effect of ApsNPFR was not long-lasting compared to ApsNPF. We still detected a significant reduction in the transcript level of ApsNPF at 36 hpi (t_4,3.33_ = 0.76, *p* < 0.05), whereas the transcript level of ApsNPFR was significantly lower only up to 24 hpi (t_4,3.51_ = 0.97, *p* < 0.05).

### 3.5. Effect on Feeding Behavior after ApsNPF and ApsNPFR Silencing

The variation in probing and feeding behavior duration was studied via EPG after RNAi-mediated silencing of sNPF and sNPFR as treatment and dsGFP as a control group. We chose 19 and 14 EPG parameters related to probing (Table 1) and phloem activities (Table 2), respectively. The activity of the stylet to reach the phloem was delayed in the dssNPF and dssNPFR treatment groups compared to the dsGFP control group (*p* < 0.001). Nevertheless, the number of total stylet probes prior to arriving at the phloem did not differ between the control and treatment groups (*p* = 0.183). The number of probes and total probing time were significantly lower in the treatment groups compared to the control group (*p* = 0.011 and *p* < 0.001, respectively). Resultantly, the period of the no-phloem stage significantly increased in the treatment groups (*p* = 0.045).

As aphids are phloem-sucking insects, the initial insertion of the stylet in the phloem was delayed from ~2.2 h (dsGFP group) to 3.5 h and 2.9 h in the dssNPF and dssNPFR group, respectively, as mentioned by “Time from start of EPG to 1st E” (*p* = 0.009). The complete period of E, E1, and E2 waves was decreased in the treatments (*p* = 0.011, *p* = 0.046, and *p* = 0.001, respectively). The number of E1 and E2 waveforms also decreased significantly in the treatments (*p* = 0.027, *p* = 0.031, respectively). Likewise, mean duration of E2 and longest E2 were reduced in the treatments (*p* = 0.036, and *p* = 0.020, respectively).

### 3.6. Effect of sNPF Silencing on Aphid Reproduction and Survival

**Figure 7:**
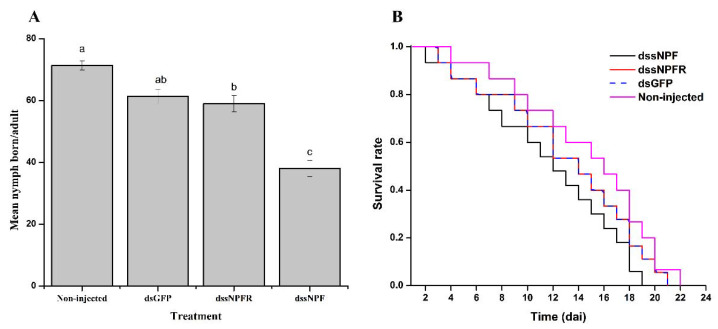
Influence of sNPF silencing on *A. pisum* reproduction and survival. (**A**) Reproduction rates in the sNPF group and the two control groups. The bars correspond to the average of three independent biological replicates. Different letters on bars indicate the statistical difference at the 0.05 level. (**B**) Survival analysis by Kaplan–Meier log-rank analysis showed no difference in survival between the sNPF treatment group and the control group (*p* > 0.05). Acronym used on X-axis: dai (days after injection).

After injecting dssNPF, dssNPFR, and dsGFP, we observed and recorded the aphid reproduction rate and survival until the progeny ceased. The maximum reproduction was seven and eight nymphs per day in the dsGFP and non-injected control groups, whereas maximum reproduction was five and seven nymphs per day in the dssNPF and dssNPFR treatments, respectively. The total numbers of N1 nymphs was significantly lower in the dssNPF treatment, namely 38 per adult, compared to the control, with 61 and 71 per adult in the dsGFP and non-injected group, respectively. However, the total number of N1 nymphs was 59 in the dssNPFR treatment. (F_3,8_ = 14.6, *p* < 0.001: Figure 7A).

The first dead aphid in the dssNPF and dssNPFR treatments and dsGFP control sample was observed on the second day after the microinjection, but there was no mortality in the non-injected group. Kaplan–Meier survival analysis showed that the dsGFP, dssNPFR, and dssNPF groups did not differ significantly in overall survival. However, the cumulative survival rate was reduced in the dssNPF group compared to the dssNPFR, dsGFP, and non-injected groups (*p* > 0.05: Figure 7B).

## 4. Discussion

The current study elucidates the sNPF precursor and predicted sNPF receptor characterization and function in an important model insect and crop pest, *A. pisum*. We amplified the *ApsNPF* peptide, predicted *ApsNPFR* cDNA sequence, and studied feeding behavior by applying RNAi bioassay. Furthermore, we observed the effect of sNPF and sNPFR silencing on aphid reproduction and survival.

There is significant variation among the neuropeptides that originate from the sNPF family. Hence, we identified that the gene structure encoding the sNPF peptide is greatly variable. Consequently, the number of introns interrupting the coding sequence range from two to four across the different insect species. Similarly, the number of exons among different insect species varies from three to five. We also compared the sNPF isoforms with other hemipterans (*Myzus persicae* and *N. lugens*), coleopterans (*T. castaneum* and *Aethina tumida*), and hymenopterans (*Apis mellifera* and *Camponotus floridanus*), which encode a single form of sNPF. In contrast, lepidopterans (*B. mori* and *Spodoptera frugiperda*) encode three (sNPF1-3), and dipterans (*D. melanogaster* and *Aedes albopictus*) encode four (sNPF1-4) isoforms derived from the identical sNPF precursor (Figure 1C). However, we discovered a consistent feature: the intron position is located after the N-terminal signal peptide in the observed species. Although a previous study suggested that holometabolous insects typically have longer sNPF precursors than hemimetabolous insects, as well as multiple sNPF isoforms [7], this does not seem to be true for all holometabolous.

The spatial distribution of sNPF and sNPFR in *A. pisum* was very high and restricted in the CNS, but the receptor was also detected in the antennae, midgut, and integument. Over the different developmental stages of *A. pisum* tested, the sNPF precursor and receptor were present in all stages of nymphs and adults, with a higher expression in first instar nymphs. This transcript profile is identical to that of *Drosophila*, where enormous neurons denoting sNPF existed in the CNS of the larva and adult stage [48]. However, our findings contradict those reported for *Glossina morsitans*, where an absence of sNPF and sNPFR expression was found in the larval instars due to differences in larval feeding behavior [49]. At the cellular level in *Drosophila*, peptides of sNPF were found to be colocalized in a wide array of neurons, and these neurons ramify in the neuropil cites of the larval CNS [9]. As Nagata et al. [29] and Root et al. [30] documented, the role of sNPF is in feeding initiation and food-seeking behavior. We assume that the detection of *ApsNPF* and *ApsNPFR* in all stages indicates their role in regulating food initiation, growth, and development, particularly in the early instars.

Intriguingly, expression of sNPF was not detected in the *A. pisum* midgut in this research, denoting that endocrine cells of the midgut do not produce sNPF (or if so, rarely), which is similar to *Drosophila* adults [27,48] and *S. gregaria* [12]. However, these findings contrast those reported for *Periplaneta americana* and *A. gambiae*, where bountiful expression was detected in the midgut because numerous sNPF colocalized with nerves compared to former species [46,50].

The highest expression of the *ApsNPF* receptor was detected in the CNS, which is identical to pre-existing observations [6,11,13,28,50]. It was surprising that expression of *ApsNPFR* was also found in the antennae, midgut, and integument, and this is similar to *D. melanogaster* and *B. mori*, although it contradicts observations in *S. gregaria* [11,13,48]. Further study of sNPFR in *Drosophila* revealed that olfactory receptor neurons (ORNs) have axons that extend from their antennae, which terminate in the glomeruli, and sNPFR was immunostained in the ORNs of the antennae [51]. As expected, bountiful expression of sNPFR was detected in the CNS and antennae of *Drosophila* [30]. We believe that these observations suggest that sNPF plays a role as a neuromodulator [7] to shape the olfactory behavior of *A. pisum*, which is similar to conclusions of previous studies [8,13,17]. These observations indicate that sNPF plays a hormonal role in digestion and olfaction.

Starvation is one of the common stresses that stimulate olfaction and locomotion to facilitate foraging behavior and the acquisition of nutrients for survival [52]. Interestingly, sNPF expression and starvation correlated differently in different species. The expression pattern of sNPF and sNPFR was upregulated during starvation stress in the CNS along the entire body, which indicates that the transcript levels depend on the nutritional state of the insect. The observation regarding the starvation-induced expression of sNPF and sNPFR in the brain contrasts with *B. mori* and *S. gregaria* [10,12,13], where the transcript profile of both genes was downregulated in response to starvation. The correlation between starvation stress and transcript expression may be due to the difference in the physiology of feeding behavior in the mentioned species. Starvation induces transcript expression of sNPF and sNPFR in *A. pisum*, which is similar to the other two dipterans, *D. melanogaster* [30] and *B. dorsalis* [17]. Our results showed that sNPFR was detected in the CNS, antennae, and midgut, which indicates that sNPF signaling not only exhibits a starvation-induced property but can also play a role as a neural modulator in *A. pisum* in response to starvation, similarly to *Drosophila* [52].

The primary documented role of sNPF discovered within the physiology of insects is feeding. To identify the function of sNPF signaling in *A. pisum*, we knocked down sNPF and its receptor. We studied feeding behavior using the EPG technique by means of dssNPF and dssNPFR injection. The silencing of sNPF and its receptor decreased the probing duration and delayed the period of phloem sap ingestion. This revealed that the sNPF signaling cascade regulates aphid feeding. Our results are in agreement with those reported for *Drosophila* and *Bactrocera*, where sNPF peptides increased the hunger behavior towards feeding [17,30,52]. Another interesting observation with *Drosophila* sNPF peptides is that they can modulate the feeding rate and affect insulin-like peptides (DILP) in growth regulation. Feeding assays instantiated that gain-of-function sNPF flies showed higher food intake, and overexpression produced bigger and heavier flies than loss-of-function sNPF-RNAi congeners [27,53]. Similarly, in *A. mellifera* and *B. mori*, the family of sNPF peptides stimulated food-searching or feeding behavior and acted as a stimulatory peptide [28,29]. However, all of these observations contrast with *S. gregaria* and *Aedes aegypti*, where sNPF signals inhibited the feeding process and RNAi-mediated knockdown of sNPF signals haphazardly increased their feeding in these species [13,31].

Finally, silencing of sNPF affected feeding in pea aphids, as well as their reproduction, but it did not reduce survival. It might be that sNPF silencing lasted for a shorter period or nutritional deficiency was not strong enough to cause death but affected reproduction only. In *Rhopalosiphum padi*, the sNPF silencing period increased the death rate in response to pesticide exposure and decreased adult longevity [20]. Previously, Will and Vilcinskas [54] observed that aphids sacrifice their reproduction ability to survive a condition of low/no nutrition availability.

As an important outcome of this project, our data explicitly linked sNPF/sNPFR signaling and feeding. Therefore, we believe the results advance our current knowledge of the sNPF/sNPFR signaling cascade and its place in regulating feeding behavior in insects. In turn, this research may contribute to the design of new strategies to control aphids, with a focus on the sNPF system.

## 5. Conclusions

In this project, we characterized sNPF and its receptor in the pea aphid *A. pisum* and discovered that sNPF was expressed at high levels in the CNS, whereas sNPFR was detected in CNS, midgut, and antennae. In addition, there was a starvation-induced expression, indicating that the transcript levels depend on the insect’s nutritional state and may stimulate locomotory behavior to obtain food. Indeed, the EPG recordings with dsRNA against sNPF and sNPFR confirmed the regulation of food uptake and feeding-related behavioral processes. Hence, the RNAi assays demonstrated effects on aphid reproduction. We believe these data increase our current understanding of the feeding mechanism and its regulation in aphids, such as *A. pisum*, and provide insight into the biological role of sNPF and its receptor.

## Figures and Tables

**Table 1:** Results for the EPG variables related to probing behavior (from initiation of EPG to before reaching the phloem) of *A. pisum* in the dssNPF and dssNPFR treatments, as well as dsGFP control groups, on broad bean seedlings.

Tissue Specificity	Parameters	dsGFP	dssNPF	dssNPFR
Epidermis	Time of start of EPG to 1st probe (s)	2.91 ± 0.05 ^c^	14.41 ± 2.14 ^a^	8.20 ± 0.86 ^b^
Epidermis and mesophyll	Time from 1st probe to 1st E2 (s)	7621 ± 1424 ^b^	11786 ± 865 ^a^	10290 ± 590 ^ab^
	Duration of 1st probe (s)	2084 ± 153 ^a^	1583 ± 153 ^b^	1699 ± 97 ^ab^
	Duration of the non-probe period before the 1st E (s)	1365 ± 61 ^b^	1749 ± 120 ^a^	1688 ± 108 ^a^
	Number of probes to the 1st E	8.2 ± 0.34 ^a^	7.5 ± 0.27 ^a^	7.65 ± 0.20 ^a^
	Number of F	2.35 ± 0.35 ^a^	1.25 ± 0.23 ^b^	1.55 ± 0.32 ^ab^
	Total duration of F (s)	62.0 ± 9.6 ^a^	39.3 ± 8.9 ^a^	41.9 ± 9.5 ^a^
	Mean duration of F (s)	27.3 ± 5.1 ^a^	23.6 ± 5.8 ^a^	19. 8 ± 5.2 ^a^
All tissues	Time from 1st probe to 1st sustained E2 (>10 min) (s)	8298 ± 1530 ^b^	13649 ± 772 ^a^	11078 ± 630 ^ab^
	Number of probes	12.35 ± 0.33 ^a^	10.8 ± 0.40 ^b^	11.05 ± 0.40 ^b^
	Total probing time (s)	28617 ± 43 ^a^	23157 ± 733 ^b^	24121 ± 453 ^b^
	Number of short probes (C < 3 min) (s)	5.69 ± 0.47 ^a^	6.47 ± 1.24 ^a^	6.13 ± 0.41 ^a^
	Number of C	10.4 ± 0.83 ^a^	9.45 ± 0.54 ^a^	9.75 ± 0.56 ^a^
	Total duration of C (s)	9847 ± 932 ^b^	12631 ± 809 ^a^	12443 ± 829 ^a^
	Mean duration of C (s)	847 ± 37 ^a^	734 ± 43 ^a^	757 ± 40 ^a^
	Number of np	9.15 ± 0.78 ^b^	12.7 ± 1.22 ^a^	12.1 ±1.05 ^ab^
	Total duration of np (s)	3054 ± 298 ^a^	3920 ± 397 ^a^	3717 ± 359 ^a^
	Mean duration of np (s)	264 ± 28 ^a^	403 ± 50 ^a^	364 ± 88 ^a^
	Total duration of no phloem phase (s)	18729 ± 981 ^b^	22248 ± 1025 ^a^	21661 ± 1119 ^a^

Abbreviations used in the second column: s (seconds). Results are shown as mean ± SE. Different lowercase letters in the same row indicate significant difference at *p* < 0.05 level by LSD test.

**Table 2:** Comparison of feeding behaviors related to phloem activity of *A. pisum* in the dssNPF and dssNPFR treatments, as well as dsGFP control groups, on broad bean seedlings.

Tissue Specificity	Parameters	dsGFP	dssNPF	dssNPFR
Epidermis and mesophyll	Time from start of EPG to 1st E2 (s)	8151 ± 1600 ^b^	12703 ± 754 ^a^	10633 ± 619 ^ab^
All tissues	Time from start of EPG to 1st E2 (s)	9005 ± 1646 ^b^	13724 ± 816 ^a^	11194 ± 824 ^ab^
	Time from start of EPG to 1st sustained E2 (> 10 min) (s)	9615 ± 1744 ^b^	14424 ± 821 ^a^	13411 ± 583 ^a^
Phloem	Number of E1	4.60 ± 0.29 ^a^	3.55 ± 0.25 ^b^	3.70 ± 0.31 ^b^
	Number of E2	4.35 ± 0.31 ^a^	3.15 ± 0.34 ^b^	3.35 ± 0.34 ^b^
	Number of single E1	0.60 ± 0.11 ^b^	1.15 ± 0.18 ^a^	1.00 ± 0.12 ^ab^
	Number of sustained E2 (> 10 min)	2.40 ± 0.19 ^a^	1.75 ± 0.19 ^b^	1.95 ± 0.15 ^ab^
	Total duration of E (s)	10530 ± 1014 ^a^	6025 ± 1014 ^b^	7563 ± 1085 ^b^
	Total duration of E1 (s)	265 ± 28 ^a^	184 ± 20 ^b^	191 ± 26 ^b^
	Total duration of E2 (s)	11674 ± 1301 ^a^	5788 ± 1031 ^b^	7181 ± 1021 ^b^
	Mean duration of E1 (s)	65.8 ± 4.33 ^a^	59.9 ± 2.90 ^a^	63.9 ± 1.79 ^a^
	Mean duration of E2 (s)	5933 ± 467 ^a^	2872 ± 1020 ^b^	3412 ± 1008 ^b^
	Duration of 1st E (s)	5172 ± 347 ^a^	2842 ± 519 ^b^	3228 ± 1021 ^ab^
	Duration of the longest E2 (s)	8543 ± 1236 ^a^	4096 ± 1027 ^b^	5112 ± 1146 ^b^

Abbreviations used in the second column: s (seconds). Results are shown as mean ± SE. Different lowercase letters in the same row indicate significant difference at *p* < 0.05 level by LSD test.

## Data Availability

The data presented in this study are available on request from the corresponding author.

## Supplementary Materials

The following supporting information can be downloaded at https://www.mdpi.com/article/10.3390/insects13030282/s1; Table S1: Primers used for qRT-PCR in this study, Table S2: Accession number of genes used for exon-intron gene structural comparison, Table S3: Accession number of genes used for phylogenetic analysis, Table S4: The Ct values of qRT-PCR result obtained from the fed and starvation experiment. Table S5: Ct values of qRT-PCR obtained from the spatiotemporal expression and gene silencing experiment, Figure S1: Reverse transcription-polymerase chain reaction (RT-PCR) analysis of starvation with sNPF (A) and sNPFR (B) compared with the expression of ribosomal protein L7 (RPL7).
