# Supplementary material for: Short Neuropeptide F and Its Receptor Regulate Feeding Behavior in Pea Aphid (Acyrthosiphon pisum)
Source: Insects. 2022 Mar 13;13(3):282. doi: 10.3390/insects13030282 (PMC8950018; doi:10.3390/insects13030282)
Supplement: Supplementary file 1 [file insects-13-00282-s001.zip › insects-1594955-supplementary.pdf]

insects MDPI

Supplementary Materials

Short Neuropeptide F and Its Receptor Regulate Feeding Behavior in Pea Aphid (*Acyrthosiphon Pisum*)

Muhammad Bilal Amir, Yan Shi, Hehe Cao, Muhammad Yasir Ali, Muhammad Afaq Ahmed, Guy Smagghe and Tong-Xian Liu

Table S1. The specific primers used in qRT-PCR and dsRNA synthesis.

Usage	Primer Name	Primer Sequences
qRT-PCR	sNPF-F	GCAGCCGTTGTGTGTACTCT
qRT-PCR	sNPF-R	CGGAGATCTCTGGTTTTTGC
qRT-PCR	sNPFR-F	GTGGCTGAACGAGAACTTCC
qRT-PCR	sNPFR-R	AAGCCGGTGCAGTCTGTAGT
qRT-PCR	RPL7-F	GCGCGCCGAGGCTTAT
qRT-PCR	RPL7-R	CCGGATTTCTTTGCATTTCTTG
dsRNA synthesis	sNPF-F	**TAATACGACTCACTATAGGG**TGGACTACGAGAATGCCAAAG
dsRNA synthesis	sNPF-R	**TAATACGACTCACTATAGGG**CCTTAACCTCAGCGACGGA
dsRNA synthesis	sNPFR-F	**TAATACGACTCACTATAGGG**AAACGCTGTTGCCCACG
dsRNA synthesis	sNPFR-R	**TAATACGACTCACTATAGGG**CACTTCGACTGCATCCTGATT
dsRNA synthesis	GFP-F	**TAATACGACTCACTATAGGG**TGACCACCCTGACCTAC
dsRNA synthesis	GFP-R	**TAATACGACTCACTATAGGG**TTGATGCCGTTCTTCTGC

The bold capital letter shows the T7 RNA polymerase promoter sequence.

Table S2. Accession numbers of the sequences used for the gene structure analysis.

**Species name**	**mRNA**	**Protein**	**Genome**
Acyrthosiphon pisum	XM_003247202.3	XP_003247250.1	NC_042494.1
Myzus persicae	XM_022314068.1	XP_022169760.1	NW_019102905.1
Nilaparvata lugens	XM_022328849.1	XP_022184541.1	NW_019105702
Drosophila melanogaster	NM_165316.2	NP_724239.1	NT_033779.5
Aedes albopictus	XM_029872461.1	XP_029728321.1	NW_021838233.1
Bombyx mori	NM_001134257.1	NP_001127729.1	NW_004581726.1
Spodoptera frugiperda	XM_035582761.1	XP_035438654.1	NC_049720.1
Tribolium castaneum	XM_008200483.2	XP_008198705.1	NC_007425.3
Aethina tumida	XM_020019114.1	XP_019874673.1	NW_017853357.1
Apis mellifera	XM_003250107.4	XP_003250155.1	NC_037641.1
Camponotus floridanus	XM_011260741.2	XP_011259043.1	NW_020229547.1

Table S3. Accession numbers of the sequences used for phylogenetic relationship analysis.

Species name-sNPF	Accession number
Acyrthosiphon pisum	XP_003247250.1
Apis mellifera	XP_003250155.1
Bombus bifarius	XP_033296993.1
Osmia lignaria	XM_034317951.1
Camponotus floridanus	XP_011259043.1
Aphis craccivora	KAF0767496.1
Drosophila melanogaster	NP_724239.1
Bactrocera dorsalis	KY652383
Zeugodacus cucurbitae	XM_011192244.2
Eumeta japonica	GBP76345.1
Bombyx mori	NP_001127729.1
Manduca sexta	XM_030172318.1
Spodoptera littoralis	JQ646093.1
Helicoverpa armigera	XM_021333218.1
**Species name-sNPFR**	**Accession number**
Acyrthosiphon pisum	XM_029489088.1
Bombus terrestris	XM_020864331.1
Apis mellifera	XM_006561685.3
Harpegnathos saltator	EFN78163.1
Solenopsis invicta	AAY88918
Bombyx mori	NM_001134235.1
Tribolium castaneum	XP_966794
Nilaparvata lugens	XM_039439868.1
Drosophila melanogaster	NP_524176
Bactrocera dorsalis	KY652384.1
Mus musculus	NP_963909.2
Oreochromis niloticus	XM_025909417.1
Oncorhynchus kisutch	XM_020463106.2
Danio rerio	AAZ04130.1
Loa loa	XM_020451317.1

Table S4. Table represents the Ct values of qRT-PCR obtained in fed and starvation experiment. Reverse transcription-polymerase chain reaction (RT-PCR) analysis of starvation with sNPF (A) and sNPFR (B) compared with the expression of ribosomal protein L7 (RPL7).

Sample	Gene	Ct value			
		Fed	3 h starvation	Fed	6 h starvation
Adult	sNPF	28.18	27.75	28.56	26.47
	RPL 7	23.18	24.30	23.78	24.13
CNS	sNPF	27.95	26.94	28.47	25.31
	RPL 7	23.26	23.86	24.26	23.69
Adult	sNPFR	28.27	27.70	28.61	26.85
	RPL 7	23.92	24.61	25.44	25.77
CNS	sNPFR	28.29	27.61	28.41	26.29
	RPL 7	23.55	24.42	23.65	23.90

Table S5. Ct values of qRT-PCR obtained from spatio-temporal expression (**A**) and gene silencing experiment (**B**).

**Ct value-A**					
Gene	Antennae	Brain	integument	Embryos	Midgut
sNPF	31.08	23.06	30.17	26.44	32.62
RPL7	19.34	18.51	20.87	18.46	18.35
	1st Instar	2nd Instar	3rd Instar	4th Instar	Adult
sNPF	22.64	25.67	25.29	26.34	24.57
RPL7	18.60	20.29	19.34	19.85	18.25
	Antennae	Brain	integument	Embryos	Midgut
sNPFR	26.29	23.25	26.14	28.06	26.03
RPL7	18.77	18.11	17.29	17.50	18.46
	1st Instar	2nd Instar	3rd Instar	4th Instar	Adult
sNPFR	23.05	26.24	26.07	25.81	25.27
RPL7	18.39	20.50	18.84	19.85	19.10

**Ct value-B**						
Time	6 hours	12 hours	24 hours	36 hours	48 hours	72 hours
sNPF	26.88	27.92	27.18	24.83	20.42	20.93
RPL7	21.01	21.33	20.84	18.45	14.56	15.54
Time	6 hours	12 hours	24 hours	36 hours	48 hours	72 hours
GFP	19.70	19.83	20.57	20.48	21.74	21.92
RPL7	13.87	14.66	15.27	14.84	16.37	16.77
Time	6 hours	12 hours	24 hours	36 hours	48 hours	72 hours
sNPFR	23.34	27.29	27.45	27.46	27.32	27.47
RPL7	10.42	15.02	15.91	15.57	17.12	18.24
Time	6 hours	12 hours	24 hours	36 hours	48 hours	72 hours
GFP	27.60	27.31	27.29	27.34	27.27	26.83
RPL7	14.97	15.61	16.12	15.67	17.13	17.42

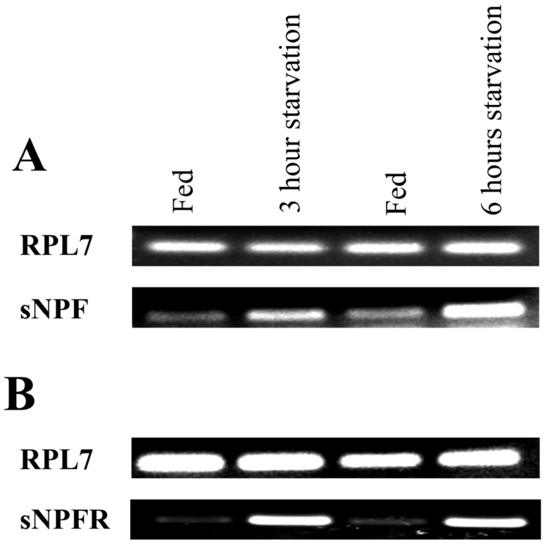

Figure S1. Reverse transcription-polymerase chain reaction (RT-PCR) analysis of starvation with sNPF (**A**) and sNPFR (**B**) compared with the expression of ribosomal protein L7 (RPL7).
